# Resilience improvement through a multicomponent physical and cognitive intervention for older people: the DanzArTe emotional well-being technology project

**DOI:** 10.1007/s40520-023-02678-3

**Published:** 2024-03-15

**Authors:** Marina Barbagelata, Wanda Morganti, Emanuele Seminerio, Antonio Camurri, Simone Ghisio, Mara Loro, Gianluca Puleo, Babette Dijk, Ilaria Nolasco, Claudio Costantini, Andrea Cera, Barbara Senesi, Nicola Ferrari, Corrado Canepa, Carlo Custodero, Alberto Pilotto

**Affiliations:** 1 Department Geriatric Care, Neurology and Rehabilitation, Galliera Hospital, Genoa, Italy; 2 Department of Informatics, Bioengineering, Robotics and Systems’ Engineering (DIBRIS), University of Genova, Genoa, Italy; 3 Foundation “Fondazione Piemonte dal Vivo”, Turin, Italy; 4 Ligurian Health Agency, Memory Clinic, Chiavari, Italy; 5 Nursing Home “Cardinal Minoretti”, Genoa, Italy; 6 Nursing Home “RSA Debouchè”, Nichelino, Turin, Italy; 7 Department of Italianistics, Romanistics, Antiquities, Arts and Performing Arts, University of Genova, Genoa, Italy; 8 Department of Interdisciplinary Medicine, “Aldo Moro” University of Bari, Bari, Italy

**Keywords:** Resilience, Multidimensional Prognostic Index, Nursing home, Older people, Multicomponent interventions, Gerontechnology

## Abstract

**Background:**

Resilience is a crucial component of successful aging. However, which interventions might increase resilience in older adults is yet unclear.

**Aims:**

This study aims to assess the feasibility and the physical and psychological effects of a technology-based multicomponent dance movement intervention that includes physical, cognitive, and sensory activation in older people living in community-dwelling and nursing home.

**Methods:**

DanzArTe program consists of four sessions on a weekly basis, using a technological platform that integrates visual and auditory contents in real time. 122 participants (mean age = 76.3 ± 8.8 years, 91 females = 74.6%) from seven nursing homes and community-dwelling subjects were assessed, before and after the intervention, with the Resilience Scale-14 items (RES-14), the Multidimensional Prognostic Index (MPI), the Psychological General Well-Being Index (PGWBI-S), and the Client Satisfaction Questionnaire-8 (CSQ-8). Mann–Whitney and Wilcoxon signed-ranks tests were used for statistical analyses.

**Results:**

At baseline significant differences in MPI and RES-14 between community-dwelling and nursing home residents were observed (*p* < 0.001 for both analyses). After the intervention, resilience significantly increased in total sample (RES-14 mean T1 = 74.6 Vs. T2 = 75.7) and in the nursing home residents (RES-14 mean T1 = 68.1 Vs. T2 = 71.8). All participants showed high overall satisfaction for DanzArTe program (CSQ-8 mean = 23.9 ± 4.4). No differences in MPI and PGWBI-S were observed.

**Discussion:**

DanzArTe was a feasible intervention and high appreciated by all older adults. Nursing home residents revealed improvements in resilience after DanzArTe program.

**Conclusion:**

The DanzArTe technology-based multi-component intervention may improve resilience in older people living in nursing homes.

## Introduction

Successful aging, defined as a multidimensional balance between different factors including illness avoidance, high physical and mental functioning, active life engagement and and psycho-social factors, has attracted greater attention with the increase of life expectancy [1, 2]. However, aging-related physical and physiological impairments inevitably affect psychological and physical well-being. A key element for older people to adapt flexibly to the different challenges of aging is resilience, i.e., the ability to cope up with adverse situations and to adjust to change [1, 3, 4].

According to literature, multicomponent personal factors influencing older subjects’ resilience are hope towards the future, life satisfaction, self-esteem, social networks, and cognitive and functional abilities [3, 5–7]. Moreover findings concerning psychological well-being and clinical data showed that high levels of resilience and low multidimensional frailty associate with better quality of life and functional status both upon admission to hospital and at discharge [1, 7].

Given this background, a recent meta-analysis suggested that multicomponent interventions based on the Comprehensive Geriatric Assessment (CGA), including physical activity, cognitive training, lifestyle education and diagnostic and therapeutic monitoring are more effective to counteract frailty and improve older people’s functional and cognitive conditions than single-domain interventions [8]. Moreover, some reviews reported interventions for resilience [9, 10] including studies with varying definitions of resilience, length of intervention without an operational construct of resilience to apply it in the clinical setting. Indeed, one study demonstrates that a predominantly group-based resilience training intervention for 12 weeks was feasible in older women with breast cancer and effective in improving resilience [11].

Additionally, a combination of physical, cognitive, leisure and cultural activities can improve aspects related to resilience, such as Quality Of Life (QoL) and psycho-social well-being in community-dwelling older people [12]. The World Health Organization (WHO) recognised the arts’ positive influence on health: cultural activities can help the wellbeing of multimorbid people [13].

Moreover, interventions in resilience should be described from a multidisciplinary and multidimensional perspective foreseeing the following aspects: (i) mindfulness meditation, helping to regulate emotions; (ii) cognitive reframing, promoting events’ reinterpretation; (iii) sense of mastery, including self-awareness, and the ability to react to situations; (iv) physical activity; and (v) social inclusion, tackling older people isolation [14, 15].

Recent studies demonstrated that also dance programmes and physical treatments based on real-time multimedia stimulation improve QoL, physical and cognitive performances, both in nursing home and community-dwelling [16–19].

In this context, the DanzArTe project is a technology-based multicomponent intervention that includes physical, cognitive, and sensory activation through dance exercises, real-time movement sonification and cognitive reframing from visual art works. This study aims to assess the feasibility and the physical and psychological effects of the DanzArTe program both in community-dwelling and nursing home older residents.

## Materials and methods

### Study design

The DanzArTe is a pre-post interventional study testing the feasibility and the effects of technology-based multicomponent dance movement intervention in multi-site and multi-centre settings.

Participants were recruited from three cultural associations (Group 1) and seven nursing homes (Group 2), starting from January through October 2022. Inclusion criteria were: age over 65 years old, the ability to provide informed consent and the absence of any motor impairments. The exclusion criteria were determined on the practical possibility of participating to the program (i.e., bedrest patients, and those who didn’t give their informed consent). The selection was made by healthcare professionals (HCP) and researchers. Before starting the enrolment of patients, an explorative phase was conducted in 20 volunteers to test the feasibility and the safety of interaction between participants and the DanzArTe technological system.

The study was performed in accordance with the Helsinki Declaration. The study was approved by the University of Genoa Ethics Committees (Protocol n.2022/11), and each participant signed an informed consent. Data were collected anonymously for the community-dwelling, by giving them a random code written on a note and asking them to put it on all the filled tests. For the nursing home residents, questionnaires’ data were by the HCPs who used a serial number to match anonymously the participant with his/her measurements.

### DanzArTe program and technological infrastructure

DanzArTe consists in the development and validation of a replicable treatment protocol for older people, including physical, cognitive, and sensory activation based on the re-enactment—in pairs or groups—of movements inspired by visual works of art. From a clinical point of view, movements encouraged by the intervention ensure the involvement in physical activity; attention and visual and auditory elaboration engage cognitive abilities. Finally, the psychophysical component is enhanced by group interaction, breaking the barrier of isolation that particularly affects the target group [20].

**Fig. 1:**
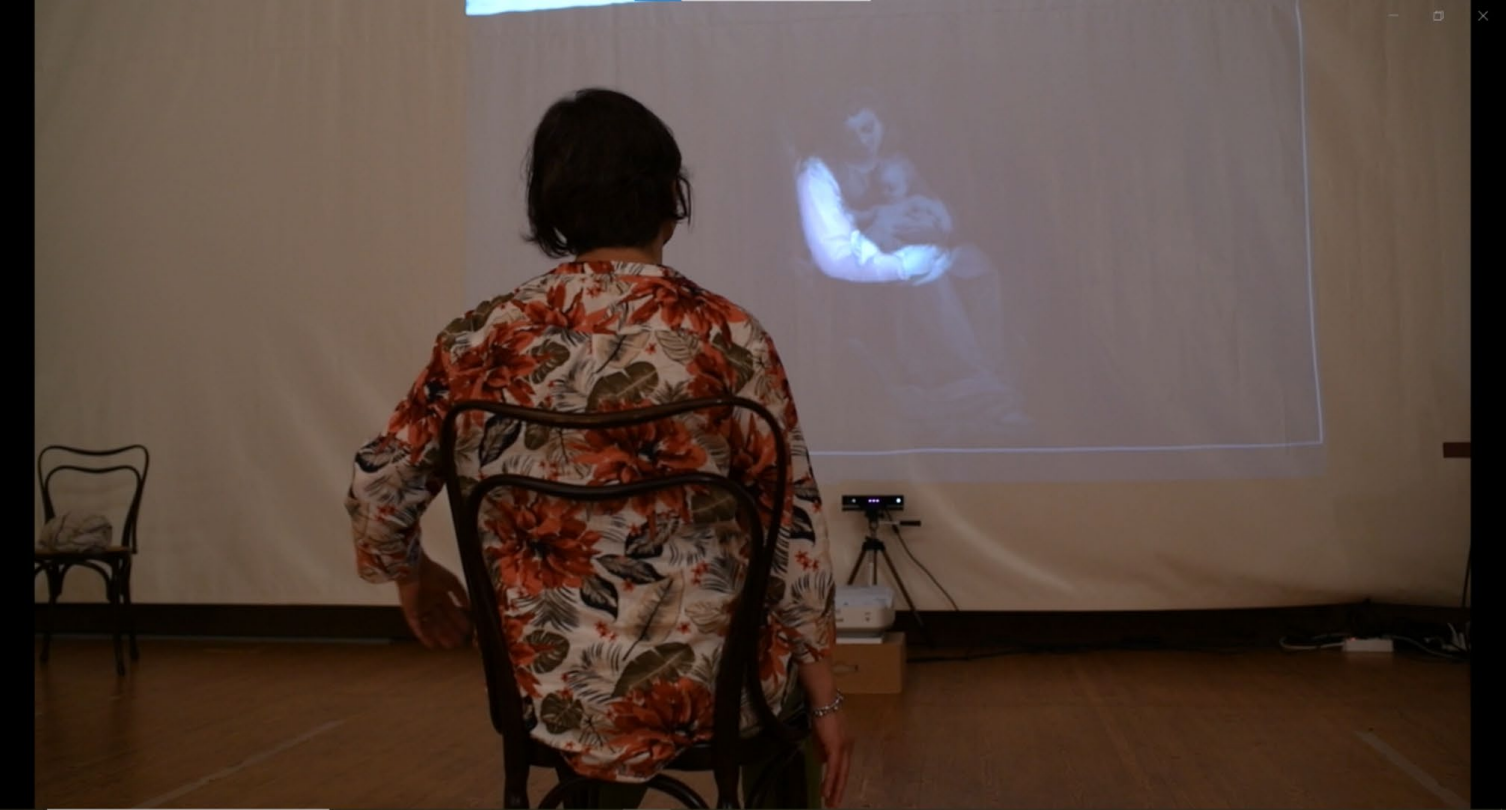
A participant engaged in the DanzArTe interaction. The painting gradually unveils as the participant moves her arm

The technological set-up consists of a scalable, affordable technology platform that enables real-time interaction with visual and sonification—*i.e.,* real-time feedback in response to users’ movement data. The intervention consists of four group sessions, each one-hour long, performed on a weekly basis, for a total of five paintings. In each session, small groups of 4–5 participants alternately emulate movements depicted in a painting showing on a video projection. Participants receive real-time feedback of their movement in the form of visual interaction (gradual unveiling of the painting) and sonification (Fig. 1). Further technical details are reported in a previous publication [21].

### Clinical and functional evaluation

DanzArTe study protocol included the administration of the following scales validated in older people: i) The Multidimensional Prognostic Index (MPI); ii) The Resilience Scale – 14 items (RES-14); iii) Psychological General Well-Being Index (PGWBI-S); and iv) Client Satisfaction Questionnaire-8 (CSQ-8).

The MPI [22] was derived from the Comprehensive Geriatric Assessment (CGA [20]) and consists of eight domains that provide data on the functional status (ADL [23]), autonomy in daily living (IADL [24]), cognitive status (SPMSQ [25]), presence of comorbidities (CIRS-CI [26]), nutritional assessment (MNA-SF [27]), risk of developing bedsores (ESS [28]), number of medications, and cohabitation status.

For each domain, the raw score was computed, and based on a tripartite hierarchy was assigned the corresponding risk group score (0 = no problems; 0.5 = minor problems; 1 = major problems). The sum of each risk category was divided by the total number of domains for which data were available. This final score can then be described in terms of three categories of risk: MPI-1 low risk for MPI values below 0.33; MPI-2 moderate risk for MPI values between 0.34 and 0.66; and MPI-3 severe risk for MPI values over 0.67. The MPI is regarded as a geriatric frailty index [6] because it is multidimensional and can predict unfavourable outcomes like falls, length of hospital stay, institutionalisation and re-hospitalisation [6, 29].

Several MPI versions have been produced over the past 15 years, the standard version and the self-administered one (Selfy-MPI [30, 31]) were employed in this study.

The RES-14 [32, 33] is the short version of the first resilience instrument developed. Using a 7-point scale, the participants indicated whether they agreed or disagreed with each of the 14 statements. Resilience scores ranged from 14 to 98, and anything under 56 is considered to be very low.

The PGWBI-S [34] is a self-administered 6-item questionnaire for evaluating health-related quality of life. Specific dimensions of anxiety, vitality, depressive mood, self-control, and positive well-being are assessed by the six PGWB-S items. Each item has 6 alternative answers (from 0 to 5) and the minimum score obtainable is 0 while the maximum is 30. 

The CSQ-8 [35]) is a validated multidimensional assessment tool of customer satisfaction with a program or service. Each question could be answered on a scale from 1 to 4, hence the possible total scores vary from 8 to 32.

### *Statistical analysis*

Socio-demographic and clinical characteristics of the individuals, of both the overall sample and the two sub-samples (Group 1—Community-Dwelling Vs. Group 2—Nursing Homes), were summarized using medians and interquartile range (IQR) for continuous variables and frequencies and percentages for the categorical ones. Firstly, gender and age differences in the two groups were checked using, respectively, the chi-square test and Mann–Whitney test. The latter test was used to evaluate differences between the two subgroups too, at the baseline, while chi-square tests were used for the comparison of frequencies between MPI risk categories. The Shapiro–Wilk test revealed violation of normality assumption (all variables had *p* < 0.032), thus nonparametric tests were carried out. Wilcoxon's signed-ranks tests were specifically used to assess DanzArTe's effectiveness on each dimension (pre- post intervention comparison). The rank biserial correlation was used to calculate the effect dimension. SPSS (version 21.0) for Mac and Jamovi were used for all analyses. A *p*-value of 0.05 or less was considered statistically significant for all two-tailed statistical tests.

## Results

### Study population

The recruited sample included 126 participants. Four of them (3.2%) were excluded due to lack of complete MPI at baseline. Thus, the final sample included 122 older subjects (mean age = 76.3 ± 8.8 years, females = 74.6%) of which 58 were community-dwelling (Group 1) and 64 nursing home residents (Group 2). Group 1 subjects were younger than Group 2 (mean age 71.3 ± 6.1 Vs. 80.9 ± 8.3 years), without gender differences between the two groups (females Group 1 = 45/58, 77.6% Vs Group 2 = 46/64, 71.9%).

### Baseline data

Table 1 shows the baseline parameters of participants as a whole and sub-divided into the two groups. As expected, community-dwelling participants (Group 1) showed a statistically significant lower impairment in all of the CGA's domains and lower multidimensional frailty than nursing home residents (MPI score Group 1 = 0.13 ± 0.10 Vs Group 2 = 0.42 ± 0.18). Additionally, community-dwelling individuals demonstrated higher levels of resilience than nursing home residents (RES-14 score Group 1 = 81.3 ± 12.8 Vs Group 2 = 68.5 ± 16.9, *p* < 0.001) but no differences in health-related quality of life, as assessed by the PGWBI (mean Group 1 = 19.5 ± 4.0 Vs. Group 2 = 18.1 ± 5.0).

**Table 1:** Baseline clinical and multidimensional characteristics of participants, divided according to Group 1 (community-dwelling) and Group 2 (nursing home residents), who participated to the DanzArTe multicomponent intervention program

Parameter	Total (*n* = 122) (median, IQR)	Group 1 (*n* = 58) (median, IQR)	Group 2 (*n* = 64) (median, IQR)	*p-*value
Age (*n* = 119)	75 (69–84)	70 (66–75)	81.5 (74–86.75)	< 0.001*
Gender (*n* = 122)	91 females (74.6%)	45 females (77.6%)	46 females (71.9%)	0.469
MPI domains—category of risk				
ADL (*n* = 121)				
Low (*n*, %)	103 (85.1%)	57 (100%)	46 (71.9%)	< 0.001*
Medium (*n*, %)	14 (11.6%)	0 (0%)	14 (21.9%)	
High (*n*, %)	4 (3.3%)	0 (0%)	4 (6.2%)	
IADL (*n* = 108)				
Low (*n*, %)	66 (61.1%)	52 (89.7%)	14 (28%)	< 0.001*
Medium (*n*, %)	8 (7.4%)	6 (10.3%)	2 (4%)	
High (*n*, %)	34 (31.5%)	0 (0%)	34 (68%)	
MNA-SF (*n* = 122)				
Low (*n*, %)	64 (52.5%)	41 (70.7%)	23 (35.9%)	< 0.001*
Medium (*n*, %)	52 (42.6%)	16 (27.6%)	36 (56.3%)	
High (*n*, %)	6 (4.9%)	1 (1.7%)	5 (7.8%)	
Number of medications (*n* = 110)				
Low (*n*, %)	60 (54.5%)	44 (78.6%)	16 (29.6%)	< 0.001*
Medium (*n*, %)	20 (18.2%)	7 (12.5%)	13 (24.1%)	
High (*n*, %)	30 (27.3%)	5 (8.9%)	25 (46.3%)	
CIRS-CI (*n* = 119)				
Low (*n*, %)	29 (24.4%)	23 (41.8%)	6 (9.4%)	< 0.001*
Medium (*n*, %)	61 (51.2%)	28 (50.9%)	33 (51.5%)	
High (*n*, %)	29 (24.4%)	4 (7.3%)	25 (39.1%)	
ESS-MOB (*n* = 121)				
Low (*n*, %)	108 (89.3%)	57 (100%)	51 (79.7%)	0.002*
Medium (*n*, %)	10 (8.2%)	0 (0%)	10 (15.6%)	
High (*n*, %)	3 (2.5%)	0 (0%)	3 (4.7%)	
SPMSQ-TYM (*n* = 102)				
Low (*n*, %)	61 (59.8%)	37 (75.5%)	24 (45.3%)	0.007*
Medium (*n*, %)	33 (32.4%)	9 (18.4%)	24 (45.3%)	
High (*n*, %)	8 (7.8%)	3 (6.1%)	5 (9.4%)	
Cohabitation status (n = 122)				
Low (*n*, %)	44 (36.1%)	44 (75.9%)	0 (0%)	< 0.001*
Medium (*n*, %)	64 (52.4%)	0 (0%)	64 (100%)	
High (*n*, %)	14 (11.5%)	14 (24.1%)	0 (0%)	
MPI index (*n* = 122)	0.25 (0.125–0.438)	0.125 (0.063–0-208)	0.402 (0.286–0.563)	< 0.001*
RES-14 (*n* = 116)	78 (65–88)	82 (73.25–92)	68 (57.25–82)	< 0.001*
PGWBIS (*n* = 117)	19 (16–22)	19 (17–22.75)	18 (16–21)	0.163

*MPI* Multidimensional Prognostic Index, *ADL* Activities of Daily Living, *IADL* Instrumental Activities of Daily Living, *MNA-SF* Mini-Nutritional Assessment-Short Form, *CIRS-CI* Cumulative Illness Rating Scale-Comorbidity Index, *ESS* Exton Smith Scale, *MOB* adjusted from Barthel Mobility Index [33], *SPMSQ* Short Portable Mental State Questionnaire, *TYM* Test Your Memory, *RES* Resilience Scale-14 items, *PGWBI-S* Psychological General Well-Being Index – Short

Statistically significant *p*-values were marked with*

### Post-intervention data

Table 2 shows the comparison between the considered parameters at baseline and after the DanzArTe multicomponent intervention. After the intervention in the overall sample and in the two Groups no changes in all the domains of the CGA and the MPI score were observed. Interestingly, a significant improvement in Resilience was observed in the whole sample (RES-14 mean at T1 = 74.6 ± 16.3 Vs. T2 = 75.7 ± 15.6, Wilcoxon’s signed rank *p*-value = 0.037, *r*_rb_ = 0.26) and, more specifically, in nursing home residents (Group 2 RES-14 mean score T1 = 68.1 ± 16.7 Vs. T2 = 71.8 ± 15.5; Wilcoxon’s signed rank *p*-value < 0.001) (Fig. 1). The Wilcoxon's signed-ranks test (*W* = 150.50, *p* < 0.001) confirmed this significant improvement in resilience and the effect size computed as rank biserial correlation (*r*_rb_ = 0.650) could be interpreted as strong [36].

**Table 2:** Comparison between pre-intervention (T1) and post-intervention (T2) clinical and multidimensional data in Group 1 (community-dwelling participants, *n* = 58) and Group 2 (nursing home residents, *n* = 64)

		Group 1 (*n *= 58)				Group 2 (*n* = 64)			
Parameter	Range	*n*	Median(IQR) T1	Median(IQR) T2	*p-*value	*n*	Median(IQR) T1	Median(IQR) T2	*p-*value
RES	14–98	56	82 (72.75–92.25)	82.5 (69.75–91)	0.452	57	67 (57–82)	72 (67–81)	< 0.001*
PGWBIS	0–30	58	19 (17–22.75)	20 (18–23)	0.266	56	18 (16–21)	19 (17–21)	0.130
MPI	0.00–1.00	57	0.13 (0.06–0.19)	0.13(0.06–0.19)	0.451	63	0.38 (0.29–0.56)	0.43 (0.31–0.50) ± 0.19	0.699

MPI domains—category of risk	T1 (*n*, %)	T2 (*n*, %)	*p-*value	T1 (*n*, %)	T2 (*n*, %)	*p-*value
ADL						
Low	56 (100%)	55 (98.2%)	N.A	44 (71%)	43 (69.4%)	0.860
Medium	0 (0%)	0 (0%)		14 (22.6%)	15 (24.2%)	
High	0 (0%)	1 (1.8%)		4 (6.4%)	4 (6.4%)	
IADL						
Low	50 (89.3%)	51 (91.1%)	0.568	14 (28%)	13 (26%)	0.569
Medium	6 (10.7%)	5 (8.9%)		2 (4%)	5 (10%)	
High	0 (0%)	0 (0%)		34 (68%)	33 (64%)	
MNA-SF						
Low	41 (73.2%)	38 (67.9%)	0.484	23 (36.5%)	26 (41.3%)	0.766
Medium	14 (25%)	18 (32.1%)		35 (55.6%)	30 (47.6%)	
High	1 (1.8%)	0 (0%)		5 (7.9%)	7 (11.1%)	
N. of Medications						
Low	44 (81.5%)	44 (81.5%)	1.000	16 (30.8%)	13 (25%)	0.346
Medium	6 (11.1%)	6 (11.1%)		12 (23.1%)	14 (26.9%)	
High	4 (7.4%)	4 (7.4%)		24 (46.1%)	25 (48.1%)	
CIRS-CI						
Low	23 (42.6%)	23 (42.6%)	0.346	6 (9.8%)	8 (13.1%)	0.530
Medium	27 (50%)	25 (46.3%)		32 (52.5%)	26 (42.6%)	
High	4 (7.4%)	6 (11.1%)		23 (37.7%)	27 (44.3%)	
ESS-MOB						
Low	56 (100%)	56 (100%)	N.A	48 (78.7%)	48 (78.7%)	N.A
Medium	0 (0%)	0 (0%)		10 (16.4%)	10 (16.4%)	
High	0 (0%)	0 (0%)		3 (4.9%)	3 (4.9%)	
SPMSQ-TYM						
Low	34 (79.1%)	35 (81.4%)	0.484	24 (50%)	25 (52.1%)	0.565
Medium	7 (16.3%)	7 (16.3%)		19 (39.6%)	19 (39.6%)	
High	2 (4.6%)	1 (2.3%)		5 (10.4%)	4 (8.3%)	
Cohabitation status						
Low	43 (76.8%)	43 (76.8%)	1.000	0 (0%)	0 (0%)	N.A
Medium	0 (0%)	1 (1.8%)		63 (100%)	63 (100%)	
High	13 (23.2%)	12 (21.4%)		0 (0%)	0 (0%)	

*MPI* Multidimensional Prognostic Index, *RES* Resilience Scale-14 items, *PGWBIS* Psychological General Well-Being Index-Short, *N.A.* not applicable

Statistically significant p-values were marked with*

**Fig. 2:**
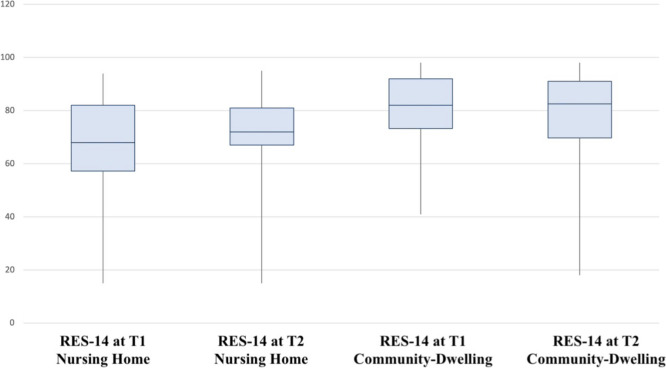
Resilience boxplots The whiskers represent minimum and maximum values while the horizontal line inside the box are the median values. The improvement in resilience score after the intervention was statistically significant for the Group 2 (Nursing home; *n* = 57) but not for the Group 1 (Community-dwelling; *n* = 56)

Figure 2 shows the boxplot for the RES-14 scale values for the two subsamples. The improvement in resilience score after the intervention was statistically significant for the Nursing Home group (*n* = 57; left side of the figure) but not for the Community-Dwelling one (*n* = 56; right side of the figure).

### Client satisfaction questionnaire-8 items

After the conclusion of the DanzArTe multicomponent intervention, 54/58 participants of Group 1 and 58/64 participants of Group 2 completed the CSQ-8. The overall population reported a median score of 24 (range population from 13 to 32, scale range 8–32). In detail, participants of Group 1 reported a median of 23 (IQR = 21–27) and participants of Group 2 reported a median value of 25 (IQR = 21–28), indicating for both the Groups high levels of satisfaction with DanzArTe multicomponent program, i.e., 72% and 78% of satisfaction in a one-hundred format of satisfaction scale for the Group 1 and Group 2, respectively.

### Post-hoc sample size analysis

Due to lack of similar study in the field, sample size analysis was conducted post-hoc according to the means and the standard deviations of the nursing home sample (*N* = 57) and to the non-normality distribution of the variables assessed. Thus, for an *α* = 0.05, two tailed and based on our sample mean, the actual power was 0.88, determined using G*power software (Kiel University, Kiel, Germany).

## Discussion

The DanzArTe program involved a total of 122 older people from two different settings (nursing homes and community-dwelling). Baseline results reported that the nursing home subjects were older and showed lower resilience, and higher multidimensional frailty compared to community-dwelling, according to previous data [7]. At the end of the DanzArTe program, the older people were evaluated through clinical scales showing the improvement in terms of resilience. As suggested in literature, intervention’s study designs for improving resilience should test the feasibility and be characterized by multicomponent features, uncontrolled trials, different samples, multi-centres and multi-site real-world settings and, mental health and satisfaction assessments [10, 12], as well as include cognitive reframing, physical activity, sense of mastery and social involvement [3, 15], as implemented in DanzArTe program.

However, we didn’t observe benefit on multidimensional frailty and QoL probably due to the brevity of the intervention. In fact, improvement in these dimensions can be more likely found in literature over a larger period [34], while intervention in resilience involving older adults seems to be effective also shorter [11].

Furthermore, older people living in nursing homes, with the lowest resilience level at baseline, are the ones who benefited more from the intervention. The same trend didn’t emerge in the community-dwelling participants probably due to their better initial resilience status.

The DanzArTe programme has been well embraced by all the participants who revealed high satisfaction, and results demonstrate both feasibility and effectiveness of a dance movement intervention for older people, guaranteeing good evidence for replicability independently from the living context.

The study has several strengths: it is the first study analyzing the effectiveness of a dance movement intervention in older people in multi-centre and multi-site settings, while few studies focused on non-pharmacological therapies in similar populations [16–19] probably due to several challenges and methodological issues of this population and settings. Therefore, DanzArTe yielded an assessment of older participants regarding the effects of an intervention designed specifically for them, contributing to overcome older’s stereotype.

Another point of strength of the study is the included cultural welfare aspect: DanzArTe is based on the interaction of participants with visual content of arts through movements and dance [16, 17]. This performance, requiring a high degree of attention, can be associated and interpreted as mindfulness meditation. Along with the cognitive activity, they were involved in dance-based physical tasks aimed to maintain a good functional state [17, 18].

The involvement of nursing home residents in DanzArTe addresses their frequent isolation by the creation of small groups of older people in a social network framework, as suggested by the literature [3–5, 15]. Moreover, despite required challenging movements and the exclusion criteria of no motor impairments, it was interesting having included this population in the study.

According to literature, longer periods of intervention are required to evaluate possible changes in frailty and quality of life: also future research could increase the duration of the entire program. At the same time, the brevity of the program was appropriate to assess and confirm the feasibility of multi-component interventions using innovative technology and addressed to older people, both in a nursing home setting and in community.

## Study’s limitations

This study has also several limitations, the lack of a control group being the primary one: in addressing this weakness, it is important to note that the present study explored the feasibility of the DanzArTe program in an uncontrolled trial study design. A RCT study design protocol will be planned as a next research step, as suggested by the literature [10].

Secondarily, the brevity of the program may have limited the possibility to observe significant changes in multidimensional parameters and QoL, as longer periods of intervention are required to have an impact on these domains. Finally, the participants were recruited only in community and nursing home settings: further studies in different contexts are needed to generalize these results of feasibility and effectiveness of the DanzArTe program.

## Conclusions

Resilience is an essential factor for a successful aging and to counteract multidimensional frailty. Multicomponent interventions are considered the most effective in improving resilience in older people. In this context, the DanzArTe programme, through works of art and dance movement, showed to be feasible, acceptable, and effective in improving resilience, especially in nursing homes residents. Despite the study limitations, this tailored approach to older people should encourage other studies and a broader implementation in clinical practice.

## Project’s partners

The program involved different institutions, representing different areas of work and functions inside the project’s framework: Casa Paganini—InfoMus, a research centre affiliated with DIBRIS (University of Genoa); Galliera Hospital, Department of Geriatric care, Neurology and Rehabilitation; Lavanderia a Vapore, Casa Europea della Danza; Museo Diocesano of Genova; RSA Galliera 'Residenza Cardinal Minoretti. In particular, we highlight the contribution of Francesca Cola, Eugenia Coscarella, Mara Forcelli, Debora Giordi, Carlotta Pedrazzoli afferent to Lavanderia a Vapore.

## Data Availability

Data supporting the conclusions of this article will be available on request from the authors.
